# Quantitative Analysis of Pyrrolizidine Alkaloids in Food Matrices and Plant-Derived Samples Using UHPLC—MS/MS

**DOI:** 10.3390/foods14071147

**Published:** 2025-03-26

**Authors:** Runfeng Lin, Jing Peng, Yingjie Zhu, Suhe Dong, Xin Jiang, Danning Shen, Jiaxin Li, Peihong Zhu, Jie Mao, Na Wang, Kun He

**Affiliations:** National Center of Biomedical Analysis, Beijing 100850, China; linrf5152@163.com (R.L.); pengjing20190603@163.com (J.P.); yingjie_zhu0130@163.com (Y.Z.); sesar_d@163.com (S.D.); jiangxzy@foxmail.com (X.J.); sdn0826@163.com (D.S.); jxin0217@163.com (J.L.); peihong_zhu@126.com (P.Z.); maojie@proteomics.cn (J.M.)

**Keywords:** pyrrolizidine alkaloids, UHPLC—MS/MS, honey, tea, milk, food safety

## Abstract

Pyrrolizidine alkaloids (PAs) are a class of nitrogen-containing basic organic compounds that are frequently detected in foods and herbal medicines. Owing to their potential hepatotoxic, genotoxic, and carcinogenic properties, PAs have become a significant focus for monitoring global food safety. In this study, an ultra-high-performance liquid chromatography–tandem mass spectrometry (UHPLC–MS/MS) method was developed for the detection and analysis of three foods (tea, honey, and milk) susceptible to PA contamination. This optimized method effectively separated and detected three types of PAs, namely, three pairs of isomers and two pairs of chiral compounds. The limits of detection (LODs) and limits of quantification (LOQs) were determined to be 0.015–0.75 and 0.05–2.5 µg/kg, respectively, with the relative standard deviations (RSDs) of both the interday and intraday precisions remaining below 15%. The average PA recoveries from the honey, milk, and tea matrices fell within the ranges of 64.5–103.4, 65.2–112.2, and 67.6–107.6%, respectively. This method was also applied to 77 samples collected from 33 prefecture-level cities across 16 provinces and included 40 tea, 6 milk, 8 honey, 14 spice, and 9 herbal medicine samples. At least one PA was detected in twenty-three of the samples, with herbal medicines exhibiting the highest total PA content. The obtained results indicate that the developed method demonstrated good repeatability and stability in the detection and quantitative analyses of PAs in food- and plant-derived samples. This method is therefore expected to provide reliable technical support for food safety risk monitoring.

## 1. Introduction

**Figure 1:**
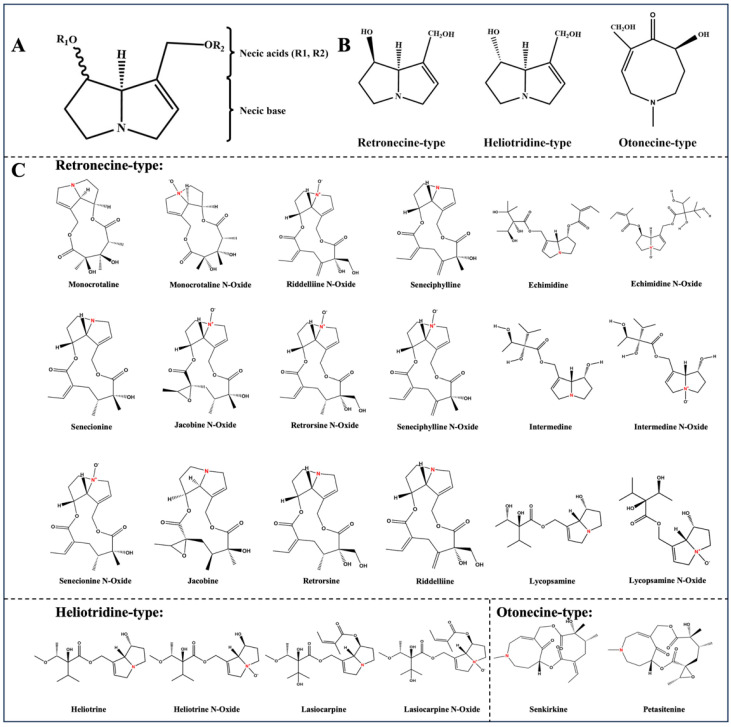
(**A**) Typical chemical structures of various PAs. (**B**) 1,2-unsaturated PAs: Retronecine type, heliotridine type, and otonecine type. (**C**) Chemical structures and classifications of 24 PAs and PANOs (isomers: jacobine, retrorsine, and senecionine N-oxide; jacobine N-oxide and retrorsine N-oxide; riddelliine and seneciphylline N-oxide. Chiral compounds: intermedine and lycopsamine; intermedine N-oxide and lycopsamine N-oxide).

Pyrrolizidine alkaloids (PAs) are secondary metabolites produced by flowering plants as a defense mechanism against herbivorous animals [1]. As one of the most widely distributed natural toxins globally [2,3], PAs exhibit significant hepatotoxicity that can lead to hepatocyte damage, hepatic fibrosis, and hepatic sinusoidal obstruction syndrome (HSOS) [4]. Additionally, some derivatives exhibit various toxic effects, including carcinogenicity, teratogenicity, and mutagenicity [5,6,7,8]. Approximately 3% of floriferous plants worldwide contain these alkaloids, which are primarily found in the families Boraginaceae (all genera), Asteraceae (e.g., the Senecio and Eupatorium genera), and Fabaceae (e.g., the Crotalaria genus) [9]. To date, more than 660 PAs and their corresponding N-oxides (pyrrolizidine alkaloid N-oxides (PANOs)) have been identified in over 6000 plant species [10]. Their typical chemical structures feature a bicyclic pyrrolizidine framework (a necine base) containing 1–2 esterified side chains (necic acids) (Figure 1A) [11]. The toxicity of any specific PA is closely related to the presence or absence of a double bond at the C1–C2 position, wherein retronecine-, inverted heliotridine-, and otonecine-type PAs bearing a double bond are highly toxic (Figure 1B), whereas platynecine-type PAs without a double bond exhibit weak toxicity or are nontoxic in nature [12,13].

In recent years, numerous studies have reported that the high levels of PA contamination in food, herbal medicines, and tea are not exclusively derived from so-called “PA-containing plants.” Instead, their presence is primarily attributable to cross-contamination during the harvesting process. Concurrently, research has demonstrated that various food-processing methods—such as pasteurization, sterilization, fermentation, and brewing—significantly influence PA concentrations [14]. For instance, some studies have indicated that pasteurization and ultra-high-temperature (UHT) sterilization do not lead to significant changes in the concentration or composition of PAs in milk, suggesting that PAs exhibit considerable stability at elevated temperatures. In contrast, during biotransformation processes such as yogurt fermentation and cheese production, the total PA content shows a marked reduction [15]. Furthermore, according to ISO guidelines and vendors’ instructions, the transfer rates of different PAs during the brewing of tea and herbal infusions vary [16]. While some PANOs are converted into free PAs during the sterilization of spices and traditional herbal medicines, the overall PA content remains essentially unchanged [17]. These dynamic changes in PA content during processing have important implications for risk assessment and food safety.

Since the first report of human poisoning from PAs in 1920, more than 17,000 cases of acute PA poisoning have been documented worldwide [13]. These poisoning incidents are closely associated with the consumption of PA-containing herbs, herbal teas, dietary supplements, or contaminated staple foods [10,18,19,20,21,22]. Given the widespread distribution of PAs and their potential health risks, international regulatory agencies have progressively strengthened the detection and regulation of relevant food products to mitigate the risk of poisoning. For example, the European Food Safety Authority (EFSA) recommends monitoring 17 key PAs in foods and feed [23], while the German Federal Institute for Risk Assessment (BfR) has established a maximum daily intake limit of 0.007 µg/kg (body weight per day) [24]. Furthermore, the European Union (EU) has updated the limit standards for PAs through Regulation (EU) 2020/2040. More specifically, the limit for teas and herbal teas intended for infants and young children (liquid) is set at 10 µg/kg, while the corresponding limit for herbal infusions (dried) and their ingredients is 200 µg/kg (calculated based on the sum of 21 PAs) [25]. However, the majority of other countries currently lack established limits for PAs in food and urgently need to enhance their regulatory frameworks to protect public health.

In recent years, the technology available for detecting PAs in complex matrices has advanced from qualitative to quantitative analyses at trace levels. Current methods include high-performance liquid chromatography with diode array detection (HPLC–DAD) [26], direct analysis through real-time mass spectrometry (DART–MS) [27], enzyme-linked immunosorbent assays (ELISAs) [28], capillary electrophoresis [29], and mass-spectrometry-based techniques such as gas chromatography–mass spectrometry (GC–MS) [30], liquid chromatography–mass spectrometry (HPLC–MS) [31,32], and high-resolution mass spectrometry (HRMS) [3,33]. Considering the high sensitivity and selectivity of the HPLC–MS and GC–MS techniques, the EFSA recommends using these two analytical techniques for the detection of PAs in foods [23,34]. However, due to the cumbersome derivatization process required for GC–MS, HPLC–MS is more widely applied because of its simpler sample pretreatment procedure [35]. Despite these developments, two significant limitations remain. Firstly, the majority of published methods often involve single detection matrices [36,37,38], thereby failing to meet the analytical demands of various food samples. Secondly, previous research has predominantly focused on retronecine- and inverted-heliotridine-type compounds, with insufficient attention being paid to otonecine-type PAs (the current research is limited to senkirkine and clivorine) [39,40,41].

Thus, in the current study, 24 PAs that are known for their high detection rates [42,43,44] and established hepatotoxicity profiles [45,46,47,48,49] were selected for analysis, encompassing three types of toxic PAs. Subsequently, a UHPLC–MS/MS method that is suitable for analyzing multiple complex matrices was developed. The sensitivity and accuracy of the method are confirmed through systematic validation of the linear range, precision (RSD < 15%), recovery (64.5–112.2%), and detection limits (0.015–0.75 μg/kg). Additionally, PA contamination levels were assessed in various commercial samples (i.e., milk, tea, honey, spices, and herbal medicines) to evaluate the method’s applicability. Overall, the aim of this study was to establish a system that provides reliable technical support for the detection and risk assessment of PAs in food.

## 2. Materials and Methods

### 2.1. Chemicals and Reagents

Reference standards with purities ranging from 95 to 99% were procured from Alta Scientific (Tianjin, China). These standards included intermedine (Im), retrorsine (Re), jacobine (Jb), lycopsamine (Ly), riddelliine (Rd), seneciphylline (Sp), echimidine (Em), monocrotaline (Mc), senecionine (Se), lasiocarpine (Lc), heliotrine (Hn), senkirkine (Sk), petasitenine (Pe), lycopsamine N-oxide (LyN), intermedine N-oxide (ImN), echimidine N-oxide (EmN), lasiocarpine N-oxide (LcN), monocrotaline N-oxide (McN), senecionine N-oxide (SeN), seneciphylline N-oxide (SpN), heliotrine N-oxide (HnN), retrorsine N-oxide (ReN), jacobine N-oxide (JbN), and riddelliine N-oxide (RdN). The types and structures of these compounds are shown in Figure 1C.

LC‒MS-grade water, acetonitrile, and methanol were supplied by Thermo Fisher Scientific (Waltham, MA, USA). Formic and sulfuric acids were supplied by Sinopharm Group Chemical Reagent Co., Ltd. (Shanghai, China). An ACQUITY UPLC HSS T3 chromatography column (100 mm × 2.1 mm, 1.8 µm), along with Oasis^®^ MCX (Mixed-mode Cation Exchange, 3 cc/60 mg) and Oasis^®^ WCX (Weak Cation Exchange, 3 cc/60 mg) cartridges, were obtained from Waters (Milford, MA, USA). PCX (3 cc/60 mg) was obtained from Agela Technologies (Tianjin, China). Ammonium hydroxide was purchased from Sigma-Aldrich (St. Louis, MO, USA).

### 2.2. Sample Collection

To ensure the diversity and representativeness of the samples, a total of 77 commercial samples were collected. Teas (n = 40), honey samples (n = 8), milk samples (n = 6), spices (n = 14), and Chinese medicines (n = 9) from different geographical locations (33 cities in 16 provinces in China) were obtained from supermarkets or online shops (Table S1). In addition, milk, honey, and tea samples were purchased from markets in Beijing and used as blank samples. The solid samples were crushed and sifted through a 50-mesh sieve. All samples were stored at 25 °C.

### 2.3. Sample Preparation

The homogenized samples (1.0 ± 0.1 g) were placed in a 50 mL polyethylene centrifuge tube. The samples were then extracted for 15 min via shaking in a 2% solution of formic acid in water (10 mL). Using a high-speed refrigerated centrifuge, the resulting mixtures were subsequently centrifuged for 10 min at a rotational speed of 10,000 rpm and at 30 °C. Each supernatant was then transferred into a new 15 mL polypropylene tube through a 0.22 µm filter membrane (hydrophilic PTFE, 13 mm, Shimadzu, Kyoto, Japan) and subjected to solid-phase extraction (SPE).

**Figure 2:**
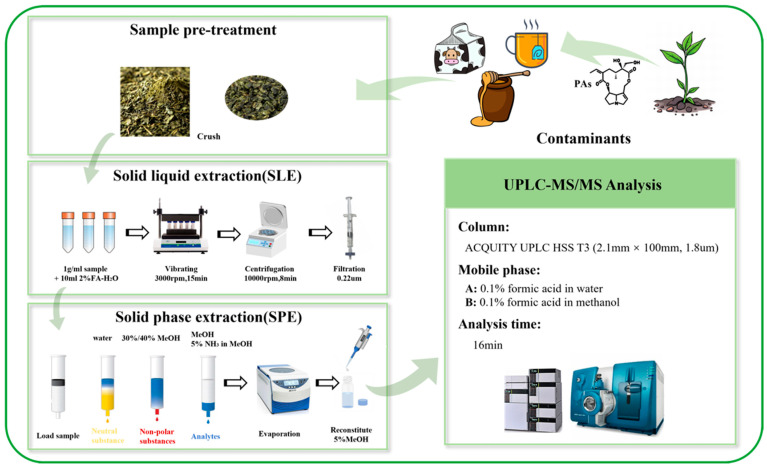
Sample preparation procedure for the determination of 24 PAs in honey, milk, and tea samples.

The SPE cartridge was initially conditioned with methanol (2 mL) and equilibrated with water (2 mL). The supernatant (0.5 mL) was subsequently loaded onto an SPE cartridge and allowed to pass through at a flow rate of 1–2 mL/min. The cartridge was then washed with water (2 mL) and 30 or 40% methanol (2 mL). Elution was performed using methanol (1 mL) and a 5% solution of ammonia methanol (1 mL); the resulting eluents were combined. The eluted mixture was concentrated using a centrifugal concentrator at 30 °C and subsequently redissolved in 5% methanol (0.1 mL). The resulting solution was subjected to centrifugation prior to LC–MS/MS analysis. The sample preparation process is illustrated in Figure 2.

### 2.4. Preparation of the Matrix-Matched Calibration Standards

Individual PA reference standards were initially prepared as 100 µg/mL primary stock solutions in either methanol or acetonitrile, with the solvent being selected based on the compound’s solubility. A standard mixed working solution containing 24 PAs (at a concentration of 1 µg/mL each) was freshly prepared using methanol and water (5:95, *v*/*v*). The calibration solutions were prepared via stepwise dilution with the same solvent to achieve concentrations of 0.05, 0.1, 0.5, 1, 5, 10, 50, and 100 µg/L. Matrix-matched calibration standards were generated by incorporating known quantities of the mixed stock solutions into appropriate volumes of uncontaminated blank matrix extracts. The blank matrix extracts were prepared according to the method outlined in the Sample Preparation Section.

### 2.5. HPLC Analysis

Twenty-four PAs were identified in the samples using a UHPLC system (LC-30AD, Shimadzu, Kyoto, Japan) in conjunction with a triple-quadrupole mass spectrometer (SCIEX QTRAP 6500, AB SCIEX, Singapore). The separation process was performed using an ACQUITY UPLC HSS T3 column (2.1 mm × 100 mm, 1.8 µm, Waters, Milford, MA, USA) maintained at 40 °C. The mobile phases were solvent A (water containing 0.1% formic acid) and solvent B (methanol containing 0.1% formic acid). The total analysis time was 16 min, the flow rate was 0.3 mL/min, and the injection volume was 3 µL. The gradient elution program was as follows: 0–1 min, 5% B; 1–10 min, 5–80% B; 10–14 min, 80% B; 14–15 min, 80–5% B; and 15–16 min, 5% B.

MS was performed in the multiple reaction monitoring (MRM) mode. The optimal collision energies (CEs) and MRM parameters for the precursor ions and product ions, along with the declustering potentials (DPs) of all target analytes, are listed in Table 1. MS was conducted in the positive electrospray ionization (ESI) mode with nitrogen as the drying and atomization gas. The analytical parameters were as follows: source temperature = 500 °C, curtain gas pressure = 25 psi, collision gas = medium setting, GAS 1 = 55 psi, GAS 2 = 55 psi, and ion spray voltage (positive polarity) = 5500 V.

### 2.6. Method Validation

To validate the analytical method in accordance with the United States Food and Drug Administration (US FDA) guidelines [50], a comprehensive evaluation of the method’s performance was conducted, including assessments of its linearity, limit of detection (LOD), limit of quantitation (LOQ), accuracy, and precision. The method was validated using three food matrices, namely, honey, milk, and tea.

Calibration curves were constructed by plotting the concentrations of the targeted PAs on the horizontal axis and their corresponding peak areas on the vertical axis. Linear regression analysis was performed for each PA by applying inverse weighting (1/x) to minimize heteroscedasticity. The resulting regression equations and coefficients of determination (R^2^) were used to assess linearity.

The matrix effect was evaluated by comparing the responses of the PAs in matrix-matched solvents (A) and in a water/methanol (95:5, *v*/*v*) mixture (B) at equal concentrations. The matrix effect was calculated according to the following equation: Matrix effect = (A/B − 1) × 100%. A matrix effect is considered strong if the value is below −40% or above +40%, whereas it is classified as weak if it falls within the range of −20 to +20% and moderate if it lies between −40 and −20% or between +20 and +40%. A positive matrix effect value leads to an enhanced signal, whereas a negative matrix effect value leads to signal suppression.

The LODs and LOQs were determined based on signal-to-noise ratios of 3 and 10, respectively. The signal-to-noise ratios were calculated using the quantitative ion pairs for each analyte. These parameters were used to determine the sensitivity of the proposed method.

To evaluate the accuracy and precision of the method, three blank matrices (honey, milk, and tea) were spiked with mixed standard solutions at three concentrations: low (1 × LOQ), medium (5 × LOQ), and high (10 × LOQ). Recovery rates were calculated to assess accuracy using the following formula:

$$Recovery\left(\%\right)=\frac{Measured\;Concentration}{Spiked\;Concentration}\times 100\%$$

Precision was expressed as the relative standard deviation (RSD), calculated as follows:

$$RSD\left(\%\right)=\frac{Standard\;Deviation}{Mean}\times 100\%$$

For each concentration level, six replicates were analyzed (n = 6) across three independent experimental batches per day over three consecutive days, resulting in 54 samples (n = 3 × 3 × 6). The intraday and interday RSDs were used to evaluate the repeatability and reproducibility of the method.

This validation process ensured that the method met the required performance criteria for linearity, sensitivity, accuracy, and precision, rendering it suitable for the reliable quantification of PAs in various food matrices.

## 3. Results and Discussion

### 3.1. Optimization of the Chromatographic Separation Conditions for the 24 PAs

**Figure 3:**
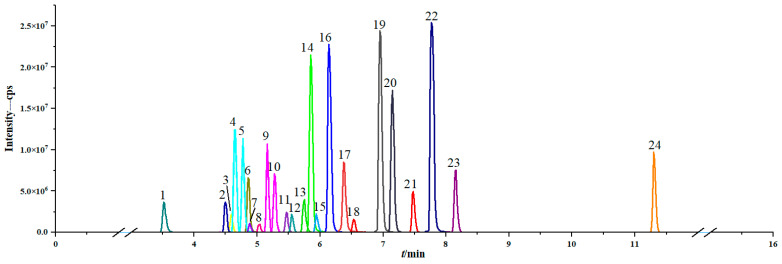
UPLC–MS/MS chromatograms of the 24 PAs/PANOs. (1, monocrotaline; 2, monocrotaline N-oxide; 3, jacobine; 4, intermedine; 5, lycopsamine; 6, jacobine N-oxide; 7, riddelliine; 8, riddelliine N-oxide; 9, intermedine N-oxide; 10, lycopsamine N-oxide; 11, retrorsine; 12, retrorsine N-oxide; 13, seneciphylline; 14, heliotrine; 15, seneciphylline N-oxide; 16, heliotrine N-oxide; 17, senecionine; 18, senecionine N-oxide; 19, echimidine; 20, senkirkine; 21, echimidine N-oxide; 22, lasiocarpine; 23, lasiocarpine N-oxide; 24, petasitenine).

Given the significant diversity in the chemical structures, molecular weights, and polarities of the target analytes, achieving the simultaneous and rapid detection of PAs poses a considerable challenge. To address this, both the chromatographic column and the mobile phase were meticulously optimized to enhance separation efficiency and sensitivity. More specifically, the separation efficiencies of two different types of columns were compared, namely, a Waters ACQUITY UPLC BEH C18 column and a Waters ACQUITY UPLC HSS T3 column. The C18 column is characterized by a wide pH range (pH 1–12) due to its trifunctionally bonded bridged ethylene hybrid (BEH) particles, excellent low-pH stability, and extremely low levels of column leakage. The T3 column features a fully porous silica packing material characterized by low ligand density and a trifunctionally bonded C18 chemistry. Additionally, it incorporates a proprietary end-capping technology, enabling the column to operate effectively with a 100% aqueous mobile phase while retaining both polar and nonpolar compounds. When using acetonitrile as the mobile phase, both columns effectively separated the PAs and thus yielded clear chromatographic peaks. However, the retention ability of the T3 column for polar compounds was significantly enhanced compared to that of the C18 column, thereby prolonging the retention times of polar targets and enhancing the separation effect. Therefore, the T3 column was selected for further analysis owing to its superior performance in resolving complex mixtures. The mobile phase was optimized by comparing methanol and acetonitrile as the solvents. Methanol provided higher peak area response values and improved resolutions (Figure S1A) and so was selected for further optimization. It was found that the diluting solvent significantly influenced the peak shape of the earliest-eluting compound, monocrotaline (RT = 3.45 min). Thus, various concentrations of methanol (5, 10, 20, 30, 40, and 50%) were evaluated as reconstitution solvents, and 5% methanol was found to yield a greater sensitivity and a more favorable peak shape, as evidenced by the narrower peak half-width (Figure S2B). This concentration provided the most favorable balance between sensitivity and peak resolution. Thus, in conjunction with the optimized mobile phase and dilution solvent, the T3 column facilitated the complete separation and highly sensitive detection of 24 PAs (Figure 3), thereby providing a reliable analytical method for the analysis of PAs in complex samples.

### 3.2. Selection of the SPE Conditions

In many instances, particularly for samples such as tea, a certain degree of processing is required after extraction because of elevated levels of interference [51]. SPE cartridges have been proven effective in removing neutral and acidic interference within matrices, thereby enhancing the sensitivity, detection, and qualitative analytical capabilities of alkaline compounds, specifically alkaloids [52,53]. This has contributed to their widespread application in analytical procedures. The SPE methodology employed in this study is based on the conventional sample-cleaning–elution approach, involving the utilization of the cation-exchange mechanism of protonated basic compounds (alkaloids) under acidic conditions. The elution process, in which an alkaline solvent is employed following the cleaning phase, is critical for the selective recovery of all PAs from SPE cartridges.

**Figure 4:**
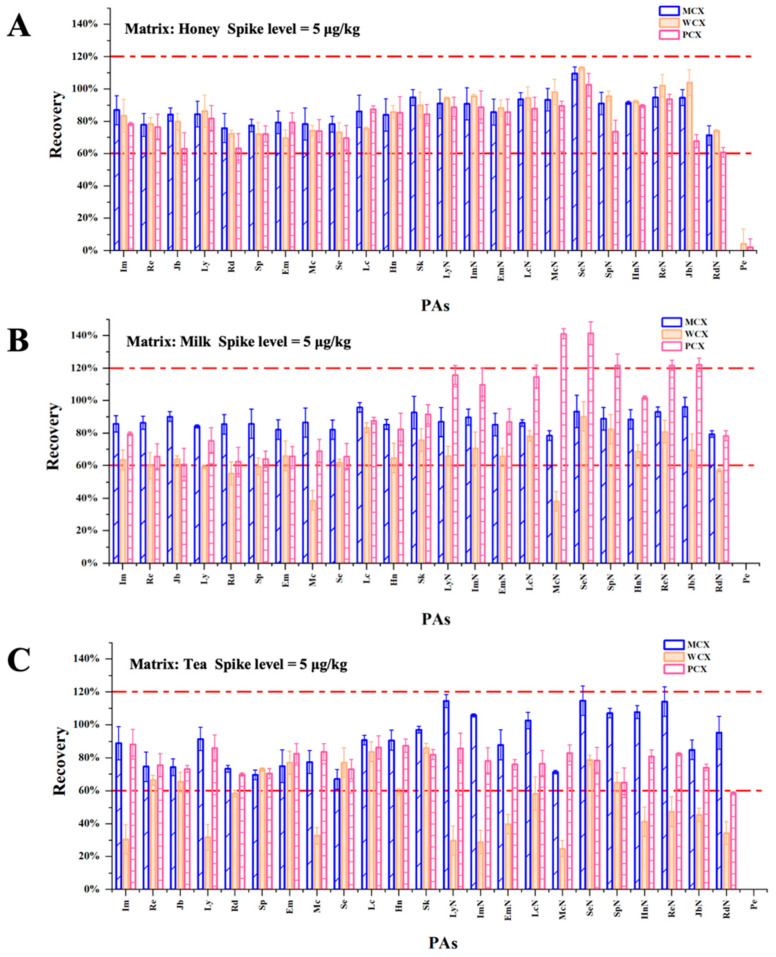
Recoveries of the 24 PAs and PANOs from the different matrices using the Cleanert^®^ PCX, Oasis^®^ MCX, and Oasis^®^ WCX cartridges and spiked levels of 5 μg/kg. (**A**) Recovery values obtained from three repeated SPE analyses of a honey sample. (**B**) Recovery values obtained from three repeated SPE analyses of a milk sample. (**C**) Recovery values obtained three repeated SPE analyses of a tea sample.

Currently, mixed-mode cation-exchange cartridges that combine ion exchange and reverse-phase retention (e.g., PCX and MCX) are commonly employed for the purification and extraction of PAs from various matrices. These cartridges have been widely applied for the analysis of soils [54], teas [55], honey samples [56], and herbs [57,58]. In this study, the purification efficacies of three types of cationic extraction cartridges (i.e., MCX, PCX, and WCX) were evaluated, with the goal being the simultaneous extraction and purification of 24 distinct PAs. The experimental procedure is described in Section 2.3. After the recoveries were calculated, a comparative analysis was performed. As illustrated in Figure 4, the MCX SPE cartridge led to superior purification in the three food matrices compared to the WCX and PCX cartridges, with the majority of the compounds exhibiting recoveries exceeding 70%. This disparity likely arose from the greater cation-exchange capacity of the MCX sulfonic acid groups (pKa < 1), which enhances the retention of PA tertiary amines under acidic loading conditions. In contrast, the carboxylate groups of the WCX cartridge (pKa ~ 5) may exhibit weaker ionic interactions, whereas the PCX cartridge is unable to effectively eliminate interference from the matrix because of its smaller pore sizes. Consequently, the MCX SPE cartridge was selected for the purification and enrichment of the 24 PAs present in the various samples. Under the optimized conditions, this SPE purification process effectively recovered the majority of PAs and eliminated potential interference from the raw extraction solution.

### 3.3. Optimization of the Rinsing Solution and Selection of the Extraction Solvent

**Figure 5:**
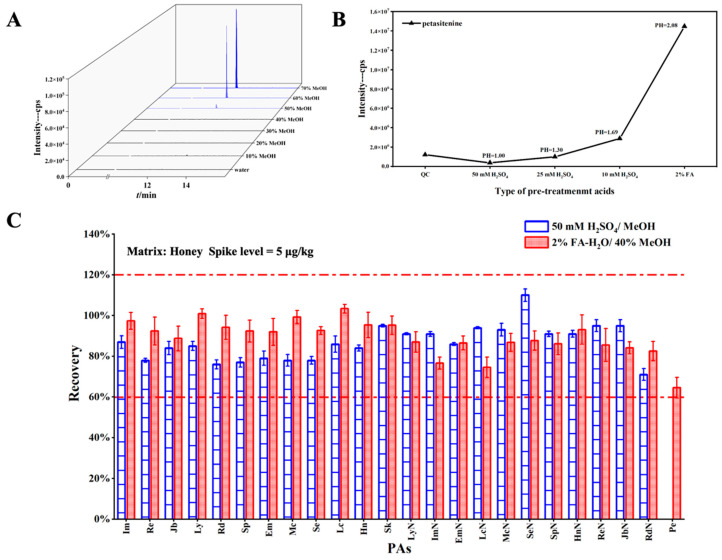
(**A**) Detection of petasitenine in the various rinsing solutions. (**B**) Detection of petasitenine following extraction with various acidic solvents. (**C**) Comparison of the PA/PANO recoveries before and after optimization.

In the experimental procedure described above, the MCX SPE cartridge was selected as the most suitable option. However, although the recoveries of most PAs met the established criteria, that of petasitenine was almost negligible. It was therefore hypothesized that the low polarity of petasitenine may have caused it to elute when it was washed with methanol. This low recovery rate raised significant concerns, as it could compromise the analytical accuracy. To resolve this issue, a stepwise elution process was employed, using various methanol/water ratios (0–70%, *v*/*v*), and the presence of petasitenine was evaluated in each eluate using LC–MS. The results indicated that a methanol concentration of ≥40% eluted petasitenine from the cartridge (Figure 5A). This finding is crucial because it demonstrates that a lower proportion of methanol in the eluent is necessary to retain petasitenine during washing.

Subsequently, the rinsing solution conditions were optimized for the honey, tea, and milk matrices. The optimization process was tailored to each matrix to account for the differences in their chemical compositions and potential interferences. Ultimately, it was determined that a 40% methanol/water solution was optimal for the honey and tea specimens, whereas a 30% methanol/water solution was optimal for the milk matrix. These optimized conditions were selected based on their ability to maximize the target compound recoveries (including petasitenine) while minimizing the matrix effects.

Additionally, based on two commonly referenced acidic extractants [59,60,61], namely, sulfuric acid and formic acid, the effects of various extractant solutions were compared, including 10 mM sulfuric acid in water, 25 mM sulfuric acid in water, 50 mM sulfuric acid in water, and 2% formic acid in water extractants. The selection of these extractants was guided by their prevalence in similar studies and ability to enhance PA extraction. It was found that the extraction efficiency of petasitenine increased upon increasing the pH of the extractant (Figure 5B), likely owing to its low polarity and the resulting interactions with the extraction medium. As shown in Figure 5C, the recovery of petasitenine from the honey matrix exceeded 60% under the optimized conditions; the recoveries of the remaining PAs continued to meet the required standards. Moreover, the recoveries of all PAs from the milk and tea matrices complied with the regulatory guidelines (Figure S2). To facilitate the simultaneous detection of all 24 PAs while ensuring their optimal recovery, a 2% solution of formic acid in water was selected as the extraction solvent. These results demonstrate that the optimized extraction and purification methods effectively address the challenges associated with the low recovery of petasitenine and ensure the accurate and reliable analysis of PAs across different matrices.

### 3.4. Method Validation

#### 3.4.1. Linearity, LODs and LOQs

To comprehensively evaluate the analytical performance of the developed method, the linear regression equations, linear ranges, and recoveries were systematically investigated at three different spiking levels, namely, low, medium, and high. These spiking levels correspond to the varying concentration ranges of the target compounds, ensuring the applicability and accuracy of the method across different concentrations. The linear range was determined by plotting the peak areas of the 24 PAs/PANOs on the vertical axis and the concentrations of the target compounds on the horizontal axis. Multiple concentration points were selected, ranging from 0.05 to 100 µg/L, to ensure both the breadth and accuracy of the linear range. The results indicate there is a strong linear relationship between the peak area and the concentrations of the 24 PAs over the tested concentration ranges (Table S2), with coefficients of determination (R^2^) ranging from 0.9920 to 0.9999. This demonstrates that the developed method exhibits excellent linearity and can allow reliable quantitative analysis across a wide concentration range.

To further verify the sensitivity of the method, low concentrations of standard substances were added to the blank matrix, and the LODs and LOQs were determined for the 24 PAs in various matrices after pretreatment. More specifically, for the honey samples, the LODs for the 24 PAs ranged from 0.015 to 0.30 µg/kg, while the LOQs ranged from 0.05 to 1.00 µg/kg (Table 2). For the tea samples, the LODs varied from 0.03 to 0.75 µg/kg, and the LOQs ranged from 0.1 to 2.5 µg/kg (Table S3). For the milk samples, the LODs ranged from 0.014 to 0.682 µg/kg, and the LOQs ranged from 0.045 to 2.273 µg/kg (Table S4).

#### 3.4.2. Recovery and Precision

**Figure 6:**
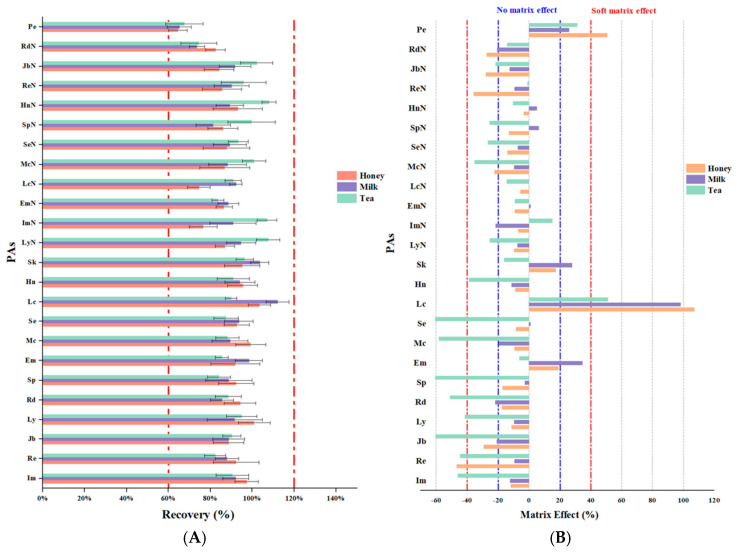
(**A**) Recoveries of the 24 PAs and PANOs from the honey, milk, and tea matrices. (**B**) Matrix effects for the 24 PAs and PANOs in the honey, milk, and tea matrices.

To comprehensively evaluate the reliability and applicability of this method in practical use, detailed recovery and precision tests were conducted using three food matrices, namely, honey, milk, and tea. Spiked recovery experiments were initially performed to validate the method at three concentration levels, including the LOQ, five times the LOQ (5 × LOQ), and ten times the LOQ (10 × LOQ). Each concentration was verified through six parallel experiments to assess the reliability of the method. The experimental data (Figure 6A) indicated that in the honey matrix, the average PA recovery ranged from 64.5 to 103.4%, with the intraday precision (RSD) ranging from 0.96 to 12.51%. In the case of the milk matrix, the recovery ranged from 65.2 to 112.2% (RSD 1.10–9.07%), while in the tea matrix, the recovery ranged from 67.6 to 107.6% (RSD 1.43–12.79%). The recoveries for these three matrices at the three spiked levels met the internationally recognized acceptable standard [50], and the precision indicators were all below the 15% threshold requirement, indicating that this method demonstrated good matrix applicability. Notably, even at the lowest spiking level (LOQ), the recoveries for all matrices exceeded 70%, with the exception of petasitenine (64.5–67.6%). Combined with the precision performance, with an RSD of <13% at each concentration level, these results confirm that the developed method offers excellent extraction efficiency and detection stability for trace component detection. Consequently, this cross-matrix analytical method provides reliable technical support for the accurate detection of PAs in complex food matrices.

To further evaluate the stability, reproducibility, and repeatability of the developed method, interday precision tests were conducted through multiple repeated experiments on different days. For the three matrices (honey, milk, and tea), the interday RSD values (n = 162) fell within the ranges of 2.80–13.89, 2.93–13.16, and 2.62–12.25%, respectively. All the interday RSD values remained within 15%, indicating that the developed method demonstrated good reproducibility and stability across repeated experiments on different days. Based on the recovery and precision test results, it was apparent that this method exhibited sufficient accuracy and reproducibility for all three food matrices.

#### 3.4.3. Evaluation of the Matrix Effect

A comprehensive investigation of the matrix effects was performed for the three food matrices (honey, milk, and tea) at both low (5 µg/kg) and high (25 µg/kg) concentrations. In Figure 6B, the results are presented as the average matrix effects across these two concentration levels. It can be seen that the majority of PAs in the milk and honey samples exhibited weak matrix effects, with the corresponding values ranging from −20 to 20%. In contrast, more pronounced matrix suppression was observed in the tea samples, suggesting that the pigments and other co-extracted compounds from the tea significantly influenced PA detection. Furthermore, lasiocarpine exhibited a strong matrix-enhancing effect across all the matrices analyzed. Based on these findings, the use of matrix-matched calibration curves is strongly recommended for the determination of PAs in various food matrices [58,62,63]. This approach effectively mitigates the interference of matrix effects on the target analytes, thereby enhancing the accuracy and reliability of quantitative analysis. Using this method, the levels of PAs can be precisely assessed in complex food matrices to provide robust support for food safety and quality control.

### 3.5. Comparison with Other Methods

In the majority of published studies [15,64], n-hexane was employed for protein precipitation during the acidic extraction of milk matrices to minimize the matrix effects and increase recoveries. In this study, the effects of an acidic extractant containing n-hexane were compared to those of an acidic extractant devoid of n-hexane in the extraction of PAs from the milk matrix. The recovery of petasitenin was significantly reduced when an acidic extractant containing n-hexane was employed, potentially due to the low polarity of petasitenine, which facilitates its migration into the organic phase during extraction. This finding suggests that traditional methods used for the extraction of PAs from milk are not suitable for PAs with lower polarities. In addition, this study made significant progress in terms of the types of detection matrices. For example, the method established by Klein et al. (2022) is applicable only to milk [65], whereas that established by Girard et al. (2023) is limited to tea [66]. In contrast, our method includes three types of food matrices, in addition to spices and herbal medicines, thereby indicating its broader applicability.

### 3.6. Analysis of Commercial Samples

The methodology employed in this study allowed the comprehensive detection and quantitative analysis of 24 PAs across 77 diverse samples. These samples included commercially available teas (7 black teas, 13 green teas, 9 oolong teas, 3 dark teas, 4 white teas, and 4 herbal teas), 14 spices, eight honey samples, six milk samples, and nine herbal medicines. The detailed results presented in Table 3 provide valuable insights into the distributions and concentrations of PAs in these samples. More specifically, in all the tea samples, PAs were detected in five samples: these PAs were echimidine, seneciphylline, lycopsamine N-oxide, and senkirkine. Notably, the concentration levels of these PAs were relatively low, with the highest total concentration being 1.5 µg/kg in one of the green tea samples. All detected levels were below the established limit standards (EU, 2020/2040), indicating a generally low contamination level in the tea samples [25]. This suggests that most commercially available teas pose a minimal risk of PA contamination. In the analyzed spice samples, PAs were identified in two instances. A particularly noteworthy finding was the detection of eight PAs and PANOs in the bitter bean powder, with a total content of 147.8 µg/kg. Although this species is not regulated (EU 2020/2040), it is worthy of attention. More specifically, it would be desirable to determine whether the elevated PA levels in bitter bean powder are due to its botanical origin or its processing methods (or both), the latter of which may facilitate the retention of these toxic compounds. Moreover, it was found that both the honey and milk samples were contaminated with a diverse array of PAs, although the overall contamination levels remained low due to the low concentrations. For instance, in the honey samples, the highest total concentration of PAs was 20.5 µg/kg, while in milk samples, the corresponding value was 28.6 µg/kg. In terms of the eight herbal medicines analyzed, various types of PAs were detected, with Senecionis scandentis hebra (1182.3 µg/kg), Farfarae flos (4618.6 µg/kg), and Arnebiae (1117.0 µg/kg) exhibiting particularly high levels of contamination. Notably, the PA and PANO concentrations in these samples exceeded EU limits (Standard 2020/2040), thereby underscoring the importance of rigorous quality control and regulatory oversight in the production and distribution of herbal medicines to mitigate the risks associated with PA contamination.

Additionally, 13 distinct types of PAs were identified in 28 commercial samples. Of these, 12 belonged to the retronecine type, while only one was classified as belonging to the otonecine type. The detection frequencies of these different PA types decreased in the following order: retrosine N-oxide (n = 21) > retrosine (n = 9) > senkirkine (n = 6) > seneciphylline (n = 5) = senecionine N-oxide (n = 5) (Figure S4). Notably, the PANO concentrations were generally higher than the PA concentrations in these specimens. This phenomenon may be attributed to the physicochemical properties of PANOs, which are hypothesized to be highly polar and readily extractable during pretreatment. This enhanced extractability therefore appeared to contribute to the higher detection rates and concentrations in the analyzed samples. Overall, the developed methodology provides valuable insights into the presence and concentrations of PAs in a wide range of samples. While the majority of samples exhibited low contamination levels, certain spices and herbal medicines (particularly Farfarae flos) were found to contain significantly higher levels of PAs. These findings highlight the requirement for continued monitoring and regulatory efforts to ensure the safety of food and medicinal products.

## 4. Conclusions

This study successfully established a highly sensitive method for the detection of pyrrolizidine alkaloids (PAs) in various food matrices. This method was based on an optimized ultra-high-performance liquid chromatography–tandem mass spectrometry (UHPLC–MS/MS) protocol. It effectively separated and identified 24 PAs, including three pairs of isomers (jacobine, retrosine, and senecionine N-oxide; jacobine N-oxide and retrosine N-oxide; riddelliine and seneciphylline N-oxide) and two pairs of chiral compounds (intermedine and lycopsamine; intermedine N-oxide and lycopsamine N-oxide). Additionally, comprehensive method validation was performed for three food matrices that are susceptible to PA contamination, namely, tea, honey, and milk samples. Furthermore, the optimized method was successfully applied to the detection of PAs in spices and herbal medicines, thereby demonstrating its broad application potential. To the best of our knowledge, this is the first in-depth analysis of petasitenine, with previous works focusing on its toxicology. Compared to the other PAs investigated in this study, petasitenine exhibited a lower polarity, and its recovery was more significantly influenced by both the pH of the extraction solution and the composition of the eluent used during sample pretreatment. This finding suggests the possibility of detecting other toxic PAs with physicochemical properties similar to those of petasitenine, which may not yet have been isolated and studied. This study therefore provides a valuable reference for future research. The method established herein was fully validated in food matrices and successfully applied for the detection and analysis of pyrrolizidine in plant-derived samples. However, despite the significant progress made in optimizing this detection method, several limitations remain. Firstly, the matrix effect that exists in complex food matrices may compromise the accuracy of the detection results, particularly in the case of lasiocarpine. Secondly, despite optimization efforts, the recovery of petasitenine remained relatively low compared to the recoveries of the other PAs. The absence of studies describing analytical methods for petasitenine may be due to the hydrolysis of its epoxide structure via ring-opening hydrolysis under strongly acidic conditions or to incomplete elution. More specifically, compared with other PAs, petasitenine has a higher pKa value, which may cause it to bind more tightly to the sulfonic acid groups on the Oasis^®^ MCX solid-phase extraction cartridge, resulting in incomplete elution under identical elution conditions. Despite these limitations, this study provides new directions for future investigations and offers a reliable technique for monitoring PAs in various foods. Future work in this area should focus on exploring the factors contributing to elevated PA levels in specific samples and developing strategies to minimize these risks. Subsequent studies should also focus on further optimizing sample pretreatment methods to mitigate the interference caused by matrix effects. Potential epoxide opening and incomplete elution of petasitenine will also be investigated by our group, and the results will be presented in due course.

## Figures and Tables

**Table 1:** Optimized MRM parameters.

Compound	Retention Time (min)	Precursor Ion [*m/z*]	Product Ions [*m/z*] (QN/QL)	DP [V]	CE [eV] (QN/QL)
Monocrotaline	3.45	326.2	120.0/237.1	70	45/35
Monocrotaline N-oxide	4.47	342.1	137.0/120.0	135	38/44
Jacobine	4.58	352.1	155.3/280.4	119	38/31
Intermedine	4.64	300.2	94.2/138.3	80	33/30
Lycopsamine	4.78	300.2	94.2/156.4	72	34/38
Jacobine N-oxide	4.85	368.2	296.2/120.3	150	34/44
Riddelliine	4.88	350.4	120.2/322.2	90	38/36
Riddelliine N-oxide	5.03	366.1	94.1/120.2	165	72/38
Intermedine N-oxide	5.16	316.2	172.0/138.0	76	38/38
Lycopsamine N-oxide	5.28	316.2	172.0/138.0	90	37/37
Retrorsine	5.48	352.1	324.2/138.2	126	37/39
Retrosine N-oxide	5.56	368.2	118.3/120.3	105	39/41
Seneciphylline	5.77	334.2	120.2/306.1	100	37/36
Heliotrine	5.87	314.2	138.0/156.1	21	29/35
Seneciphylline N-oxide	5.95	350.2	120.0/138.0	106	40/34
Helotrine N-oxide	6.15	330.2	172.0/111.0	99	36/53
Senecionine	6.40	336.2	120.0/308.2	131	40/38
Senecionine N-oxide	6.53	352.2	118.0/94.0	143	43/77
Echimidine	6.96	398.2	120.0/220.1	77	32/26
Senkirkine	7.15	366.2	168.1/150.0	106	40/36
Echimidine N-oxide	7.48	414.2	254.1/396.2	61	40/31
Lasiocarpine	7.79	412.2	120.0/336.2	30	36/27
Lasiocarpine N-oxide	8.17	428.2	254.1/410.2	100	38/32
Petasitenine	11.30	404.3	348.1/292.1	27	17/24

**Table 2:** LODs, LOQs, and precisions for detection of the 24 PAs in honey.

Analyte	LOD (µg/kg)	LOQ (µg/kg)	RSD (Intraday, %)			RSD (Interday, %)		
			LOQ	5 × LOQ	10 × LOQ	LOQ	5 × LOQ	10 × LOQ
Intermedine	0.015	0.050	4.15	10.27	2.52	13.89	11.67	5.55
Retrorsine	0.150	0.500	6.91	8.28	2.92	7.87	6.32	10.85
Jacobine	0.150	0.500	6.09	2.69	2.20	7.15	6.30	7.21
Lycopsamine	0.015	0.050	2.36	12.51	5.74	9.52	8.83	7.56
Riddelliine	0.150	0.500	5.95	7.39	6.56	11.01	10.29	7.56
Seneciphylline	0.150	0.500	5.39	7.84	2.15	9.61	9.14	8.49
Echimidine	0.015	0.050	6.62	8.08	1.72	8.57	6.02	11.62
Monocrotaline	0.150	0.500	3.24	3.54	2.14	4.56	9.48	7.15
Senecionine	0.150	0.500	1.88	8.20	2.84	4.34	7.49	5.98
Lasiocarpine	0.015	0.050	2.12	9.73	2.20	2.80	8.28	5.35
Heliotrine	0.015	0.050	6.27	18.23	4.13	6.92	10.86	7.26
Senkirkine	0.015	0.050	4.53	8.92	3.07	9.45	6.39	8.31
Petasitenine	0.300	1.000	5.14	4.85	4.82	4.87	5.39	4.44
Lycopsamine N-oxide	0.075	0.250	5.05	2.54	3.33	7.67	3.98	4.64
Intermedine N-oxide	0.075	0.250	2.94	5.88	3.82	6.16	4.06	6.56
Echimidine N-oxide	0.150	0.500	3.43	5.84	0.96	4.21	8.06	3.87
Lasiocarpine N-oxide	0.075	0.250	4.99	3.40	7.99	4.65	6.39	5.39
Monocrotaline N-oxide	0.150	0.500	4.44	3.51	3.79	11.36	11.05	11.96
Senecionine N-oxide	0.150	0.500	4.72	2.95	6.28	7.08	10.05	11.08
Seneciphylline N-oxide	0.150	0.500	5.34	2.15	4.91	5.56	5.75	7.17
Helotrine N-oxide	0.015	0.050	7.21	5.06	2.06	6.48	9.95	11.75
Retrosine N-oxide	0.075	0.250	8.09	3.97	4.33	7.05	4.31	9.35
Jacobine N-oxide	0.075	0.250	3.01	3.12	1.66	4.82	3.47	7.06
Riddelliine N-oxide	0.150	0.500	4.69	4.86	5.92	8.22	5.69	4.59

**Table 3:** Types and concentrations of PAs and PANOs detected in commercial samples.

Sample Type	Sample Number	Type of PA	Concentration (µg/kg)	Concentration of Total PAs (µg/kg)
Black tea Green tea Oolong tea	1	Echimidine	0.2	0.2
	1	Seneciphylline	0.2	1.5
	1	Seneciphylline	0.3	0.3
	2	Lycopsamine N-oxide	0.9	0.9
	3	Senkirkine	1.3	1.3
Amomum kravanh	1	Retrosine N-oxide	92.5	92.5
Bitter-bean powder	1	Retrorsine, Seneciphylline, Seneciphylline N-oxide, Senecionine, Senecionine N-oxide, Intermedine, Intermedine N-oxide, Lycopsamine	5.0, 27.4, 48.8, 17.1, 40.9, 1.5, 3.4, 3.6	147.8
Angelicae sinensis	1	Retrorsine, Retrosine N-oxide	7.1, 113.2	120.3
Codonopsis	1	Senecionine N-oxide	2.4	2.4
Senecionis scandentis hebra	1	Riddelliine N-oxide, Seneciphylline, Seneciphylline N-oxide, Senecionine, Senecionine N-oxide, Senkirkine, Retrosine N-oxide	33.7, 362.4, 621.3, 63.4, 83.8, 0.5, 17.2	1182.3
Farfarae flos	1	Senecionine, Senecionine N-oxide, Retrosine N-oxide	736.2, 3833.6, 48.8	4618.6
Ragwort	1	Lycopsamine N-oxide, Senkirkine, Retrosine N-oxide	50.9, 18.3, 23.8	93.0
Arnebiae	1	Intermedine, Intermedine N-oxide, Lycopsamine, Lycopsamine N-oxide, Senkirkine, Retrosine N-oxide	147.8, 712.0, 34.8, 196.0, 1.1, 25.2	1117.0
Eupatorii	1	Echimidine, Senkirkine, Retrosine N-oxide	0.4, 1.1, 6.9	8.4
Tu-San-Qi	1	Seneciphylline, Senecionine, Senecionine N-oxide, Echimidine, Senkirkine, Retrosine N-oxide	3.1, 4.8, 3.7, 0.4, 16.5, 22.2	50.6
Honey	1	Retrorsine, Retrosine N-oxide	3.3, 10.8	14.1
	2	Retrorsine, Retrosine N-oxide	6.2, 11.0	17.2
	3	Retrorsine, Intermedine N-oxide, Retrosine N-oxide	4.8, 0.5, 11.7	17.0
	4	Retrorsine, Retrosine N-oxide	4.1, 14.7	18.8
	5	Retrosine N-oxide	16.7	16.7
	6	Retrorsine, Retrosine N-oxide	3.2, 15.1	18.3
	7	Retrosine N-oxide	20.5	20.5
Milk	1	Retrorsine, Retrosine N-oxide	0.8, 23.5	24.3
	2	Retrosine N-oxide	22.7	22.7
	3	Echimidine, Retrosine N-oxide	0.3, 26.2	26.5
	4	Retrorsine, Retrosine N-oxide	1.6, 22.2	23.9
	5	Lycopsamine N-oxide, Retrosine N-oxide	4.6, 23.9	28.6
	6	Retrosine N-oxide	27.9	27.9

## Data Availability

The original contributions presented in the study are included in the article/Supplementary Materials, further inquiries can be directed to the corresponding author.

## Supplementary Materials

The following supporting information can be downloaded at https://www.mdpi.com/article/10.3390/foods14071147/s1. Figure S1: Optimization of the LC conditions; Figure S2: Comparison of the 24 PA/PANO recoveries before and after optimization; Figure S3: Comparison of the 24 PA/PANO recoveries when using 2% formic acid in water + n-hexane and 2% formic acid in water as extraction solvents; Figure S4: Types of PAs/PANOs detected in the commercial samples and the number of times these were detected; Table S1: Origins of the 77 commercial samples; Table S2: Method validation based on the linearity for the 24 PAs/PANOs; Table S3: LOD, LOQ, and precision for detection of the 24 PAs/PANOs in tea; Table S4: LOD, LOQ, and precision for detection of the 24 PAs/PANOs in milk.
