# Supplementary material for: Quantitative Analysis of Pyrrolizidine Alkaloids in Food Matrices and Plant-Derived Samples Using UHPLC—MS/MS
Source: Foods. 2025 Mar 26;14(7):1147. doi: 10.3390/foods14071147 (PMC11989101; doi:10.3390/foods14071147)
Supplement: Supplementary file 1 [file foods-14-01147-s001.zip › foods-3529774-supplementary.pdf]

Supplementary Materials

Quantitative Analysis of Pyrrolizidine Alkaloids in Food Matrices and Plant-Derived Samples by UHPLC–MS/MS

Runfeng Lin [#], Jing Peng [#], Yingjie Zhu, Suhe Dong, Xin Jiang, Danning Shen, Jiaxin Li, Peihong Zhu, Jie Mao, Na Wang * and Kun He *

National Center of Biomedical Analysis, Beijing 100850, China

* Correspondence: Corresponding author: Na Wang, Email: nwang@ncba.ac.cn; Kun He, Email: hk@proteomics.cn

# Runfeng Lin and Jing Peng contributed equally to this work.

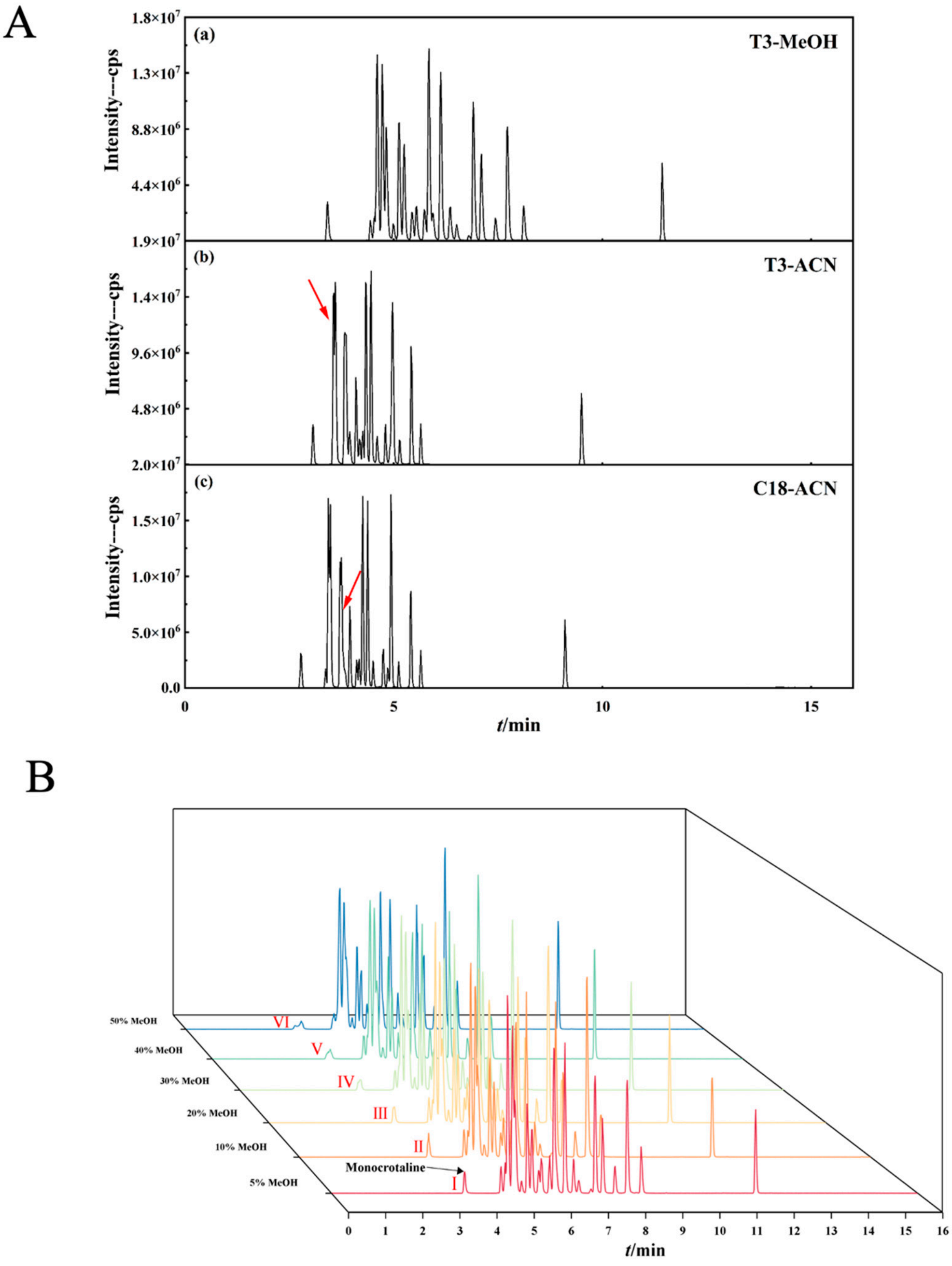

Fig. S1. Optimization of the LC conditions. (A) Chromatographic separation of the PAs under different column and mobile phase solvents (a. Column: T3, Mobile phase: MeOH-H_2O; b. Column: T3, Mobile phase: ACN-H_2O; c. Column: C18, Mobile phase: ACN-H_2O). (B) Chromatographs of the PAs under different reconstitution solvent conditions (I: 5% MeOH; II: 10% MeOH; III: 20% MeOH; IV: 30% MeOH; V: 40%MeOH; VI: 50% MeOH).

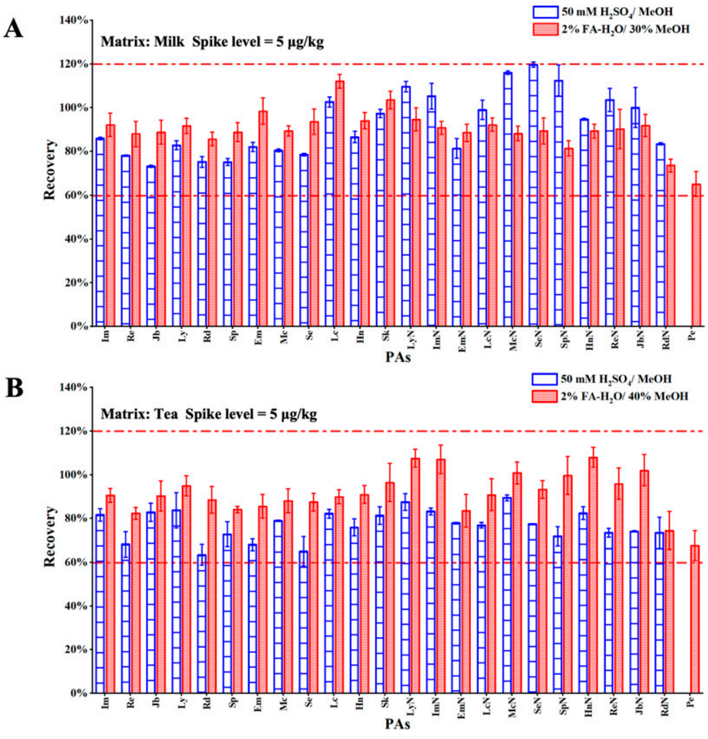

Fig S2. Comparison of the 24 PA/PANO recoveries before and after optimization. (A) Matrix: milk, Pre-optimization conditions: 50 mM H_2SO_4 and MeOH, Optimized conditions: 2% FA-H_2O and 30% MeOH. **(B)** Matrix: tea, Pre-optimization conditions: 50 mM H_2SO_4 and MeOH, Optimized conditions: 2% FA-H2O and 40% MeOH.

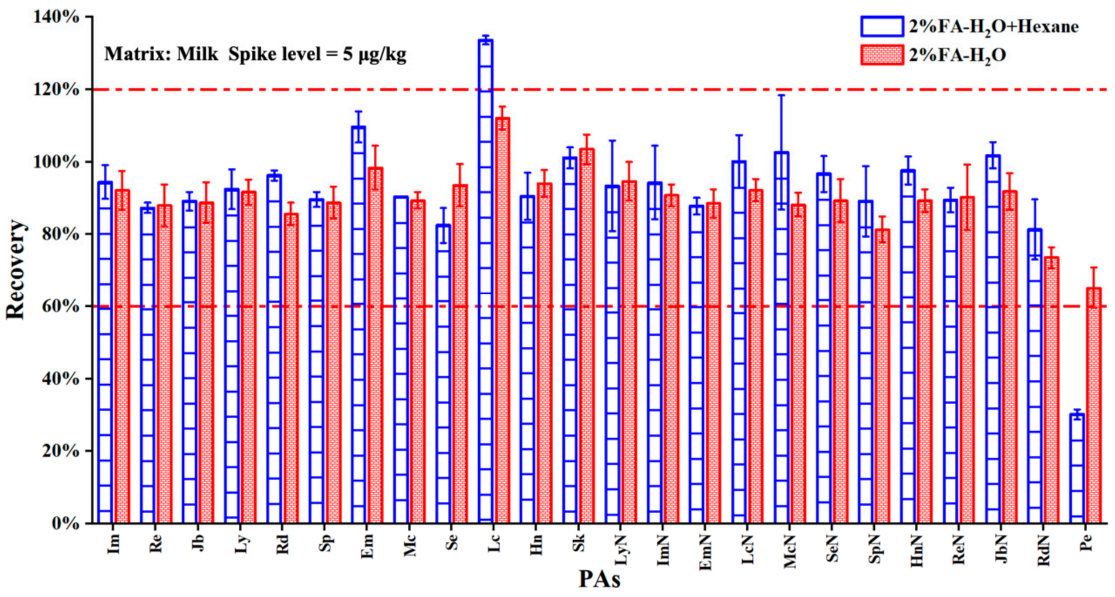

Fig S3. Comparison of the 24 PA/PANO recoveries when using 2% formic acid in water + n-hexane and 2% formic acid in water as extraction solvents.

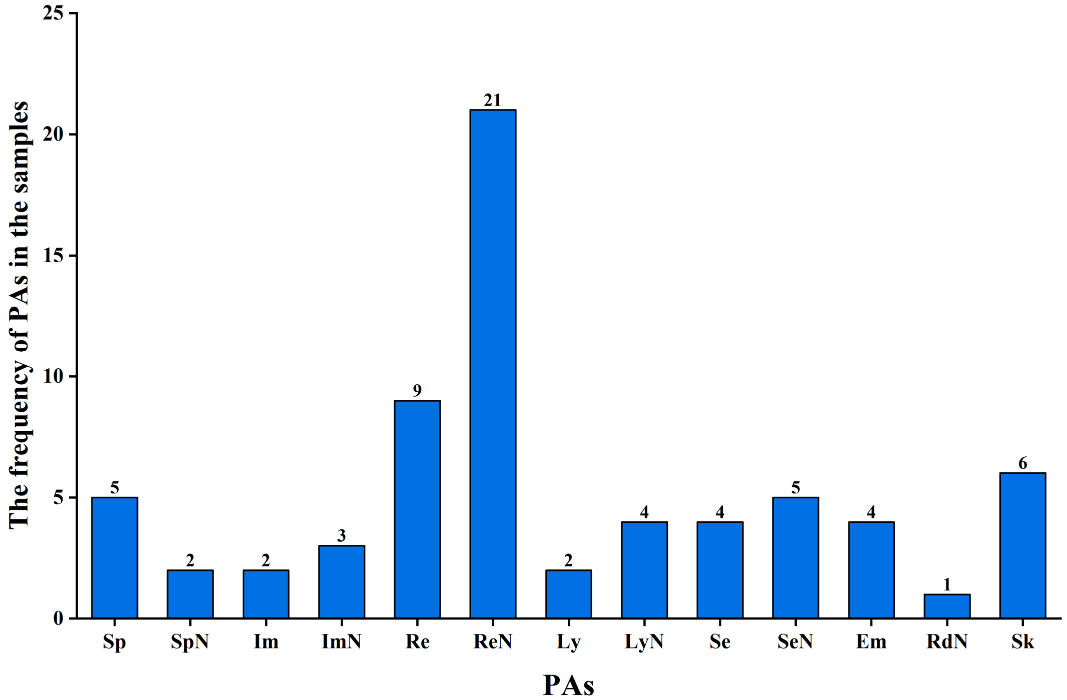

Fig S4. Types of PAs/PANOs detected in the commercial samples and the number of times these were detected.

Table S1. Origins of the 77 commercial samples

Samples	sum	Origin
Black tea	7	Huangshan, Anhui; Qiongzhong, Hainan; Guilin, Guangxi; Wuyishan, Fujian; Wuzhishan, Hainan
Green tea	13	Huangshan, Anhui; Fujian; Suzhou, Jiangsu; Qiongzhong, Hainan; Hangzhou, Zhejiang; Leshan, Sichuan; Hengxian, Guangxi; Rizhao, Shangdong; Hainan
Dark tea	3	Pu'er, Yunnan; Liuzhou, Guangxi
Oolong tea	9	Fujian; Yangzhou, Jiangsu; Quanzhou, Fujian
Herb tea	4	Zhongwei, Ningxia; Linxia, Gansu; Datong, Shanxi
White tea	4	Anji, Zhejiang; Hong Kong, China
Milk	6	Xinxiang, Henan; Tianjin, China; Beijing, China; Shijiazhuang, Hebei
Honey	8	Jinan, Shandong; Wuhan and Yingcheng, Hubei
Spice	14	Shaanxi and Gansu
Herbal medicine	9	Bozhou, Anhui

Table S2. Method validation based on the linearity for the 24 PAs/PANOs

Matrix	Analyte	Linear regression equation	Linear range (μg/kg)	R^2
	Intermedine	y = 1.15755e6 x + 20813.2	0.05–2	0.9996
	Retrorsine	y = 1.17361e5 x + 160.259	0.05–2	0.9928
	Jacobine	y = 1.65023e5 x + 1809.95	0.23–10	0.9996
	Lycopsamine	y = 1.07415e6 x + 8600.11	0.05–2	0.9984
	Riddelliine	y = 7.76886e4 x + 31634.1	0.45–20	0.9998
	Seneciphylline	y = 2.67076e5 x + 20645.0	0.23–10	0.9996
	Echimidine	y = 2.01825e6 x + 11016.7	0.05–2	0.9979
	Monocrotaline	y = 4.04115e5 x + 195527	0.23–10	0.9995
	Senecionine	y = 2.72442e5 x + 26181.7	0.23–10	0.9998
	Lasiocarpine	y = 2.21948e6 x − 10801.9	0.05–2	0.9902
	Heliotrine	y = 2.31458e6 x + 8315.31	0.05–2	0.9998
Honey	Senkirkine	y = 1.07594e6 x + 2286.25	0.05–2	0.9967
	Petasitenine	y = 2.99870e4 x − 386.260	2.5–100	0.9978
	Lycopsamine N-oxide	y = 5.51701e5 x + 10816.1	0.23–10	0.9991
	Intermedine N-oxide	y = 6.60691e5 x + 6627.78	0.23–10	0.9985
	Echimidine N-oxide	y = 2.33207e5 x + 9512.94	0.23–10	0.9993
	Lasiocarpine N-oxide	y = 3.28428e5 x + 1976.08	0.23–10	0.9991
	Monocrotaline N-oxide	y = 1.85122e5 x + 11342.5	0.23–10	0.9983
	Senecionine N-oxide	y = 1.06732e5 x + 5378.79	0.23–10	0.9979
	Seneciphylline N-oxide	y = 1.72339e5 x + 13981.5	0.23–10	0.9985
	Helotrine N-oxide	y = 2.42008e6 x + 6927.66	0.05–2	0.9989
	Retrosine N-oxide	y = 1.68478e5 x + 7144.82	0.05–2	0.9954
	Jacobine N-oxide	y = 5.52629e5 x + 14233.5	0.05–2	0.9985

	Riddelliine N-oxide	y = 8.74946e4 x + 5628.71	0.45–20	0.9992
Milk	Intermedine	y = 8.79760e5 x + 18215.3	0.05–2	0.9996
	Retrorsine	y = 8.89516e4 x + 33245.7	0.05–2	0.9928
	Jacobine	y = 1.47723e5 x + 21717.0	0.23–10	0.9996
	Lycopsamine	y = 7.98423e5 x + 30770.4	0.05–2	0.9984
	Riddelliine	y = 7.01326e4 x + 16286.8	0.45–20	0.9998
	Seneciphylline	y = 2.27237e5 x + 36258.7	0.23–10	0.9996
	Echimidine	y = 1.64184e6 x + 3.2750.8	0.05–2	0.9979
	Monocrotaline	y = 4.22021e5 x + 3.7432.8	0.23–10	0.9995
	Senecionine	y = 2.52507e5 x + 3.1940.6	0.23–10	0.9998
	Lasiocarpine	y = 1.77630e6 x + 26704.2	0.05–2	0.9902
	Heliotrine	y = 1.80758e6 x + 38720.2	0.05–2	0.9998
	Senkirkine	y = 8.02813e5 x + 12428.2	0.05–2	0.9967
	Petasitenine	y = 3.84261e4 x − 10978.0	2.5–100	0.9978
	Lycopsamine N-oxide	y = 4.31259e5 x + 45174.3	0.23–10	0.9991
	Intermedine N-oxide	y = 4.42257e5 x + 47739.7	0.23–10	0.9985
	Echimidine N-oxide	y = 2.07326e5 x + 22674.2	0.23–10	0.9993
	Lasiocarpine N-oxide	y = 2.83097e5 x + 24275.2	0.23–10	0.9991
	Monocrotaline N-oxide	y = 1.63898e5 x + 15821.7	0.23–10	0.9983
	Senecionine N-oxide	y = 8.60434e4 x + 8939.87	0.23–10	0.9979
	Seneciphylline N-oxide	y = 1.38515e5 x + 23519.6	0.23–10	0.9985
	Helotrine N-oxide	y = 2.08234e6 x + 46235.1	0.05–2	0.9989
	Retrosine N-oxide	y = 1.33100e5 x + 24723.3	0.05–2	0.9954
	Jacobine N-oxide	y = 5.46737e5 x + 11823.2	0.05–2	0.9985
	Riddelliine N-oxide	y = 8.04051e4 x + 12902.2	0.45–20	0.9992
Tea	Intermedine	y = 5.44678e5 x + 5456.01	0.1–4	0.9994
	Retrorsine	y = 8.01984e4 x + 10593.2	1–40	0.9991
	Jacobine	y = 6.73135e4 x − 7797.21	0.5–20	0.9991
	Lycopsamine	y = 4.96295e5 x + 16139.0	0.1–4	0.9993
	Riddelliine	y = 3.28416e4 x − 1284.33	1–40	0.9997
	Seneciphylline	y = 1.08675e5 x − 10926.1	1–40	0.9999
	Echimidine	y = 1.23853e6 x − 8684.48	0.1–4	0.9998
	Monocrotaline	y = 1.49349e5 x − 1934.05	0.25–10	0.9996
	Senecionine	y = 1.20741e5 x − 47575.1	1–40	0.9994
	Lasiocarpine	y = 1.02485e6 x − 10865.0	0.1–4	0.9983
	Heliotrine	y = 1.15263e6 x − 16303.6	0.25–10	0.9999
	Senkirkine	y = 5.73643e5 x − 3362.52	0.1–4	0.9987
	Petasitenine	y = 4.29289e4 x − 30764.5	2.5–100	0.9928
	Lycopsamine N-oxide	y = 2.75508e5 x + 2320.95	0.5–20	0.9995
	Intermedine N-oxide	y = 3.49610e5 x + 378789	0.5–20	0.9979
	Echimidine N-oxide	y = 1.72914e5 x + 5051.43	0.5–20	0.9995
	Lasiocarpine N-oxide	y = 2.27784e5 x − 5236.72	0.25–10	0.9998
	Monocrotaline N-oxide	y = 1.13521e5 x + 4540.49	0.25–10	0.9982

Senecionine N-oxide	y = 7.02410e4 x − 155.209	0.5–20	0.9968
Seneciphylline N-oxide	y = 1.01562e5 x + 18043.1	0.5–20	0.9975
Helotrine N-oxide	y = 1.58432e6 x + 5820.53	0.1–4	0.998
Retrosine N-oxide	y = 1.20268e5 x + 13510.3	0.25–10	0.997
Jacobine N-oxide	y = 3.07988e5 x + 19523.5	0.25–10	0.9997
Riddelliine N-oxide	y = 6.20789e4 x − 2676.66	0.5–20	0.9992

Table S3. LOD, LOQ, and precision for detection of the 24 PAs/PANOs in tea

Analyte	LOD (μg/kg)	LOQ (μg/kg)	RSD (intra-day, %)			RSD (inter-day, %)		
			LOQ	5×LOQ	10×LOQ	LOQ	5×LOQ	10×LOQ
Intermedine	0.030	0.100	3.25	10.88	6.28	4.00	12.25	7.73
Retrorsine	0.300	1.000	2.74	3.60	5.23	3.91	4.27	5.08
Jacobine	0.150	0.500	6.93	7.41	2.66	6.05	4.88	4.32
Lycopsamine	0.030	0.100	4.61	12.79	7.58	8.83	9.98	7.16
Riddelliine	0.300	1.000	6.12	5.25	6.57	6.67	5.94	6.26
Seneciphylline	0.300	1.000	1.43	4.37	4.55	4.31	4.25	5.40
Echimidine	0.030	0.100	5.44	4.17	3.32	6.17	5.35	3.17
Monocrotaline	0.075	0.250	5.49	3.91	5.53	5.40	6.51	5.62
Senecionine	0.300	1.000	4.09	4.73	4.74	3.50	5.49	5.87
Lasiocarpine	0.030	0.100	3.25	3.88	3.55	4.01	3.26	2.69
Heliotrine	0.075	0.250	4.08	7.09	6.30	3.22	11.00	7.75
Senkirkine	0.030	0.100	8.76	3.24	3.24	7.16	3.57	4.12
Petasitenine	0.750	2.500	6.79	11.10	10.76	9.49	9.65	8.87
Lycopsamine N-oxide	0.150	0.500	4.21	2.14	4.62	4.26	2.62	5.57
Intermedine N-oxide	0.150	0.500	6.50	9.15	6.60	5.91	7.67	4.70
Echimidine N-oxide	0.150	0.500	7.44	2.21	1.97	5.73	5.06	2.78
Lasiocarpine N-oxide	0.075	0.250	7.37	4.42	1.79	7.49	4.90	3.88
Monocrotaline N-oxide	0.075	0.250	4.99	3.89	4.80	5.84	4.51	5.62
Senecionine N-oxide	0.150	0.500	4.10	3.98	2.35	4.90	7.20	4.80
Seneciphylline N-oxide	0.150	0.500	8.65	12.27	10.27	8.42	10.89	11.34
Helotrine N-oxide	0.030	0.100	4.55	3.29	4.14	4.00	3.94	3.37
Retrosine N-oxide	0.075	0.250	7.19	6.03	2.89	6.07	7.60	10.61
Jacobine N-oxide	0.075	0.250	7.23	4.37	5.87	6.27	9.12	7.70
Riddelliine N-oxide	0.150	0.500	8.74	7.95	3.56	9.13	6.28	8.59

Table S4. LOD, LOQ, and precision for detection of the 24 PAs/PANOs in milk

Analyte	LOD (μg/kg)	LOQ (μg/kg)	RSD (intra-day, %)			RSD (inter-day, %)		
			LOQ	5×LOQ	10×LOQ	LOQ	5×LOQ	10×LOQ
Intermedine	0.014	0.045	5.35	4.90	3.23	8.07	6.44	6.17
Retrorsine	0.014	0.045	5.76	4.44	6.83	4.99	4.19	5.47
Jacobine	0.068	0.227	5.58	4.55	3.02	5.79	7.44	7.66
Lycopsamine	0.014	0.045	3.46	5.24	5.47	6.66	13.04	13.16
Riddelliine	0.136	0.455	3.06	7.23	4.28	5.64	5.68	5.52
Seneciphylline	0.068	0.227	4.42	5.74	3.04	5.90	8.36	10.98
Echimidine	0.014	0.045	5.99	7.47	3.85	6.67	8.75	6.47
Monocrotaline	0.068	0.227	2.30	5.47	2.30	6.42	13.11	8.58
Senecionine	0.068	0.227	5.85	5.89	3.13	7.17	7.85	6.72
Lasiocarpine	0.014	0.045	3.09	3.37	3.41	4.25	3.90	5.46
Heliotrine	0.014	0.045	3.69	4.65	3.54	6.42	7.09	7.09
Senkirkine	0.014	0.045	4.02	4.42	3.49	7.11	5.99	4.30
Petasitenine	0.682	2.273	5.60	6.47	6.07	5.04	5.92	5.77
Lycopsamine N-oxide	0.068	0.227	5.29	4.81	4.42	6.23	8.95	6.99
Intermedine N-oxide	0.068	0.227	3.01	3.30	3.64	7.33	10.30	11.10
Echimidine N-oxide	0.068	0.227	3.88	4.84	1.92	7.28	6.96	4.93
Lasiocarpine N-oxide	0.068	0.227	3.11	7.11	3.07	5.74	4.89	2.93
Monocrotaline N-oxide	0.068	0.227	3.39	1.84	1.10	6.26	8.88	8.98
Senecionine N-oxide	0.068	0.227	5.91	6.60	3.54	6.01	9.63	7.89
Seneciphylline N-oxide	0.068	0.227	3.48	3.70	2.16	7.70	6.86	8.17
Helotrine N-oxide	0.014	0.045	3.14	5.84	3.64	5.38	8.30	6.44
Retrosine N-oxide	0.014	0.045	9.07	7.52	4.12	10.68	7.03	8.29
Jacobine N-oxide	0.014	0.045	5.05	5.58	4.28	7.18	7.53	7.50
Riddelliine N-oxide	0.136	0.455	2.87	4.71	2.04	4.85	4.47	3.59
